# Intergenerational continuity of multidimensional patterns of child maltreatment exposure: A person-centered approach

**DOI:** 10.1017/S0954579425100217

**Published:** 2025-07-03

**Authors:** Justin Russotti, Jennifer Warmingham, Hannah Swerbenski, Elizabeth D. Handley, Zhi Li, Dante Cicchetti

**Affiliations:** 1 Mt. Hope Family Center, University of Rochester, Rochester, USA; 2 Columbia University, New York, USA; 3 University of Minnesota, Minneapolis, USA

## Abstract

One of the most devastating and costly consequences of CM is that it persists across generations. Yet, we know little about whether there is intergenerational continuity of diverse dimensions of CM exposure (e.g., chronicity, multi-subtype) or unique patterns of exposure. This is a critical gap, given evidence that different forms of CM confer unique consequences. To enhance our understanding of intergenerational continuity of CM, the current study applied a multidimensional framework to be the first to investigate whether unique forms of CM exposure (characterized by the subtypes and whether multi-type exposure occurred) exhibited homotypic/heterotypic patterns of intergenerational continuity. Latent class analysis (LCA) was used to identify patterns of CM exposure in mothers and their offspring (aged 8–13) who were part of a high-risk, economically disadvantaged sample of maltreated and nonmaltreated youth (*N* = 1240). Four distinct classes of CM exposure were identified in both mothers *(“Single-Subtype without Sexual Abuse”; “Sexual Abuse”, “Multi-Subtype Exposure”;* and *“No Maltreatment”*) and offspring *(“No Maltreatment”; “Single Type-Neglect”; “Single Type-Abuse”;* and *“Chronic, Multi-type”*). Patterns of homotypic and heterotypic intergenerational continuity were identified, with a pattern of multi-type exposure emerging as an enduring form of exposure across generations. Implications for preventive interventions are discussed. Intergenerational continuity of multidimensional patterns of child maltreatment exposure: A person-centered approach

## Introduction

Child maltreatment (CM)—defined by physical, sexual and emotional abuse and neglect (U.S. Department of Health & Human Services (USDHHS), 2022)—is a highly prevalent public health problem that represents one of the most adverse and stressful challenges that confronts children. Approximately 12.5% of all youth in the U.S. will experience substantiated CM in their lifetime (USDHHS, 2022). Some 618,000 children are victims of substantiated CM each year (USDHHS, 2022). CM is a broad-ranging risk factor known to potentiate compromised development and maladaptation across virtually every domain of functioning (e.g., cognitive, emotional, interpersonal, neurobiological, and physical development; Cicchetti & Toth, 2016; Widom, 2014). These deleterious consequences result in an estimated economic burden of $830,928 per victim of non-fatal child maltreatment across the lifespan (Peterson et al., 2018). One of the most devastating and costly consequences of CM is that exposure to CM is associated with continuity, or persistence of maltreatment exposure in the subsequent generation (Madigan et al., 2019). Addressing the intergenerational continuity of CM offers the opportunity to contain its destructive effects before cycles of adversity and sequelae can be entrenched.

## Intergenerational continuity of CM

Parental history of CM is a well-documented risk factor for CM exposure in offspring (Cicchetti & Rizley, 1981; Madigan et al., 2019). Parental history of child maltreatment, although not deterministic in subsequent parenting and child maltreatment risk (Assink et al., 2018), is associated with elevated rates of maltreatment exposure in the next generation directly via perpetration by the CM-exposed parent, termed “*transmission*” (Cicchetti & Rizley, 1981). Intergenerational CM “*continuity”—*the focus of the current study*—*refers to circumstances where CM exposure occurs in multiple generations, regardless of whether the CM-exposed parent perpetrated the CM (Berlin et al., 2011; Langevin et al., 2022; Berzenski et al., 2022). Results from two recent meta-analyses (Assink et al., 2018; Madigan et al., 2019) and one umbrella analysis (van Ijzendoorn et al., 2020) report modest support for the intergenerational continuity of maltreatment. Though both *transmission* and *continuity* are important areas of study, *intergenerational continuity* focuses less on the specific source of transmission (e.g., how caregivers become perpetrators) and more broadly addresses whether and how maltreating environments are recreated in subsequent generations, even when a caregiver is not the perpetrator (Noll et al., 2017). For example, mothers with sexual abuse histories may not perpetrate sexual abuse but may struggle to protect their child, fail to prevent abuse or intervene to stop abuse, and in other ways recreate environments in which abuse by *others* is allowed to persist (Noll et al., 2017). Thus, intergenerational *continuity* of CM encompasses the diversity of ways in which the offspring of CM survivors may also be exposed to CM (i.e., intergenerational *equifinality*; Cicchetti & Rogosch, 1996).

## A multidimensional approach to continuity

Thus far, studies have predominantly taken a unidimensional approach to investigate intergenerational continuity, operationalizing CM exposure as a homogeneous binary construct (exposed/not exposed; Berzenski et al., 2022). Within this model, continuity is established if parent and child each experience any form of CM, regardless of whether they experience distinct forms (i.e., parent exposed to sexual abuse and child exposed to neglect). This aggregated, undifferentiated approach to continuity of CM has helped identify parental CM exposure as a vitally important factor in understanding intergenerational patterns of caregiving – in clinical and research settings. Yet, it ultimately obfuscates the incredible heterogeneity of survivors’ lived experiences (i.e., variation in the type(s), timing, and chronicity of exposure; Jackson et al., 2019; Jonson-Reid et al., 2012; Manly, 2005) and thus has limited utility to drive targeted and effective prevention/intervention approaches.

Drawing on developmental psychopathology theory (e.g., Cicchetti & Rizley, 1981), Berzenski et al. (2013, 2022) have advocated for a more sensitive and specific *multidimensional* framework to guide research on intergenerational CM. A multidimensional framework disaggregates CM exposure, affording the opportunity to investigate how different CM features (e.g., type of CM) transcend generations (i.e., differentiated approach). Within this multidimensional lens, one can observe intergenerational patterns of a) *homotypic continuity*, defined as when a parent and child experience the same form/pattern of CM and b) *heterotypic continuity,* where there is intergenerational continuity in exposure to CM, generally, but parent and child experience different forms/patterns of CM (Rutter et al., 2006). The greater clarity afforded by a multidimensional approach to understanding intergenerational continuity of CM can improve the precision of our interventions by allowing us to understand the implications of specific patterns of parental exposure to CM on family functioning in the next generation. As Berzenski et al. (2013) aptly note, if we fail to differentiate how multiple dimensions of CM exposure influence intergenerational patterns, “we risk overlooking important implications for practice at best or misinforming prevention and intervention efforts at worst” (pp. 12).

There has been an uptick in studies applying a multidimensional lens to the intergenerational continuity of CM (see Berzenski & Yates, 2022 for review). So far, this literature has almost exclusively focused on the continuity of individual CM *subtypes* (i.e., physical abuse, sexual abuse, emotional abuse, and neglect) across generations. Current research does support modest intergenerational continuity of physical abuse and some heterotypic patterns of exposure (e.g., parental neglect to child sexual or physical abuse; Berzenski et al., 2022). These innovative studies have deepened our understanding of how individual subtypes experienced by parents may create specific or broad risk for maltreatment in the next generation. However, co-occurrence of maltreatment subtype or additional dimensions (e.g., multi-type exposure) have not yet been considered as part of investigations of intergenerational maltreatment continuity.

Characterizing intergenerational CM using a multi-subtype, multi-dimensional approach is vital to advance this literature. Subtypes of CM rarely occur in isolation, and multi-type exposure is often the norm, rather than the exception, particularly among children involved in the child protective system (Herrenkohl & Herrenkohl, 2009; Vachon et al., 2015). Likewise, CM exposure can be episodic (e.g., occurring in infancy and then not again) or chronic (e.g., occurring in two or more developmental periods), with more chronic exposure conferring more negative health outcomes (English et al., 2005; Russotti et al., 2021). Indeed, studies suggest that CM features such as chronicity and multi-type exposure have greater explanatory power than specific CM types (Smith & Pollak, 2021). Further, individuals can have vastly different CM experiences depending on how multiple CM dimensions cluster together (Warmingham et al., 2019). For example, exposure to both sexual abuse *and* chronic neglect represents a *qualitatively different* impact on development than say, experiencing episodic sexual abuse without neglect. It is likely that combinations of CM type, chronicity, and multi-type exposure act in concert to influence development, and when we separate out individual CM features for isolated analysis, we lose vital information on the phenomenon that is CM.

Therefore, additional features of CM that make individual experiences distinct (i.e., multi-type exposure) deserve attention in multidimensional models of intergenerational continuity (Berzenski et al., 2022). A small number of studies have investigated how individual CM characteristics (e.g., chronicity, multi-type exposure, timing) increased risk for intergenerational continuity (e.g., Ben-David et al., 2015; McKenzie et al., 2021; St-Laurent et al., 2019; Thornberry & Henry, 2013). However, these studies focus on how certain characteristics of the *parent’s* CM history are predictive of the child’s general maltreatment status (presence/absence; i.e., undifferentiated approach). Additionally, these studies investigate distinct CM parameters with univariate statistical approaches (e.g., multivariable regression), which do not attend to the clustering of parameters. Thus, we still know little about whether diverse CM parameters (e.g., chronicity, multi-type exposure, timing), and/or clusters of multiple CM parameters, spread across generations. And, given that studies have demonstrated that diverse forms and multidimensional patterns of CM exposure have different correlates and consequences (e.g., Berzenski & Yates, 2011; Noll et al., 2021), it is critical that we deepen our understanding of how multidimensional patterns of CM traverse generations.

## Person-centered approaches to CM exposure

Although it is difficult to simultaneously account for multiple features of CM exposure, advancements in multivariate modeling techniques have improved our ability to capture the multifaceted nature of CM (Brieant et al., 2024). While several multivariate options exist (see Brieant et al., 2024), latent class analysis (LCA) has shown promise as a viable option well-suited to illustrate the diversity and variability of CM experiences by comprehensively accounting for the typically overlapping, yet distinctly meaningful features of CM (Gabrielli & Jackson, 2019; Rivera et al., 2018). Contrasted with variable-centered techniques, which assume homogeneity within a sample and obfuscate individual-level differences, LCA is a person-centered approach that aims to reveal the unique heterogeneity within a sample. LCA uses a probabilistic modelling algorithm that allows clustering of data and statistical inference (Sinha et al., 2021).

As applied to the study of child maltreatment specifically, LCA has been used with success to identify subgroups or classes of maltreated children based on the types of maltreatment experienced (e.g., neglect, and sexual, physical, and emotional abuse). Findings consistently demonstrate a latent class, or common pattern, that is characterized by multiple subtypes of maltreatment (see Rivera et al., 2018 for review). Further, studies have begun to incorporate indicators of CM chronicity, along with type(s) and multi-type occurrence to further parse heterogeneity (Warmingham et al., 2019; Ziobrowski et al., 2020). Warmingham et al. (2019) operationalized chronicity using a single ordinal chronicity indicator defined by either CM exposure in zero, one, or two plus developmental periods, in addition to several maltreatment subtype indicators, revealing patterns of episodic, single-type exposure and a chronic, multi-type exposure. Similarly, Ziobrowski and colleagues (2020) examined multiple types of CM across two periods, childhood and adolescence, finding time-limited CM classes and a chronic multi-subtype abuse class spanning both developmental periods.

Person-centered approaches to CM exposure have advanced our understanding of individual variation in patterning of CM *within* a single generation, but the intergenerational implications of unique CM classes remain largely unexplored. To our knowledge, Negriff (2020) conducted the only study to partially address this gap, applying LCA to determine intergenerational continuity in specific patterns of adverse childhood experiences (ACEs) between parents and adolescent offspring. Negriff (2020) identified two distinct classes of ACEs exposure in parents and offspring characterized by high and low levels, revealing some presence of intergenerational continuity of adversity. Yet, to our knowledge, this approach has not been specifically applied to the intergenerational continuity of CM. CM is a unique phenomenon from ACEs, which include exposure to a broader range of adverse experiences in childhood (e.g., parental divorce or death), and thus CM is deserving of a specific focus. Parsing heterogenous patterns of CM, and then determining whether unique patterns exhibit intergenerational (dis)continuity may reveal previously indiscernible information about how varied, multifaceted forms of CM exposure progress across generations, as well as which forms do not. Such detail can inform the study of distinct intergenerational mechanisms and help optimize precious resources by tailoring family-based interventions to meaningful, vulnerable subgroups, with the hope of effectively reducing the multigenerational reach of CM.

## Current study

To enhance our understanding of the intergenerational continuity of CM, we aimed to be the first study to investigate whether latent classes of CM exposure in mothers were associated with latent classes of CM exposure in offspring. More specifically, we examined this phenomenon in a high-risk, economically-disadvantaged sample of maltreated and non-maltreated youth (children aged 8–13). Informed by Berzenski et al. (2022) multidimensional model of intergenerational CM, we explored forms of heterotypic and homotypic (dis)continuity. Our sample is specifically well-suited to address this research question because we have previously conducted exploratory (Warmingham et al., 2019) and confirmatory (Russotti et al., 2025) latent class analyses to identify patterns of child maltreatment subtype and chronicity (based on coded CPS records) for the child generation. Thus, we aim to 1) identify patterns of maternal maltreatment subtype based on maternal report of CM experiences and 2) investigate the associations between maternal CM patterns and previously identified offspring CM patterns.

We expected to identify distinct subgroups of CM exposure within the parent generation (hereafter referred to as “G1”) and among offspring (hereafter referred to as “G2”). G2 classes are based on previously published work demonstrating a set of four distinct and replicable subgroups of CM exposure in the current sample (Russotti et al., 2025; Warmingham et al., 2019). The four classes are characterized as follows: 1) *No Maltreatment*—a group of children with no documented exposure; 2) *Single Subtype Exposure*—a group of children exposed to a single subtype of maltreatment (∼90% of which was a form of abuse), mostly in one developmental period; 3) *Neglect Only*—characterized by exposure to neglect that occurred largely in a single developmental period; 4) *Multi-Subtype, Chronic Maltreatment—*a pattern of exposure typified by multi-subtype exposure (100%) that was mostly chronic in nature. We expected that patterns of CM exposure would be slightly different among G1s and G2s, given differing measurement, indicators, and developmental range of exposure. However, like the patterns identified in G2s in this sample, we expected to observe distinct G1 subgroups characterized by single-type and multi-type exposure. We also expected to identify incidences of both heterotypic and homotypic forms of intergenerational continuity. Results will lend a clearer understanding of which patterns of CM exposure are most likely to persist or desist across generations.

## Methods

### Participants

The current study was drawn from a larger sample of *n* = 1,240 children ages 8–13 (*M*_age_ = 10.42, SD = 1.31) who participated in a research-based summer camp from 2004 to 2012 (see Cicchetti et al., 1990) for further information about the research camp setting). Participants were balanced across female (*n* = 606; 48.9%) and male (*n* = 631; 50.9%), and 69.0% of the participants identified as Black, 16.4% identified as white, 8.9% identified as bi-racial, 2.3% Asian, 1.2% American Indian/Alaskan Native, and 2.2% identified as “other.” Approximately half of the participants were exposed to maltreatment of any kind (*n* = 647; 52.2%).

Participants were recruited first via a review of documented records of child abuse and neglect reports from the Department of Human Services (DHS). These Child Protective Services (CPS) records were reviewed by a DHS liaison, who identified children exposed to maltreatment. Within this identified group, the DHS liaison contacted a random sample of eligible families and explained the study to parents who consented to have their information shared with the project staff. Once shared, project study staff met with parents, who provided informed consent for their and their child’s participation in the research-based summer camp, as well as access to their DHS records.

**Figure 1.**
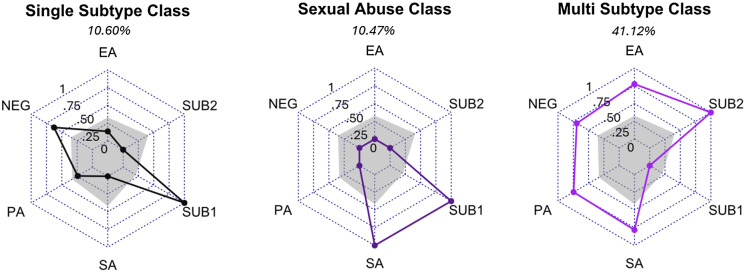
G1 maternal maltreatment classes. *Note.* item response probabilities range from 1.0 (all members of this had this subtype) to zero (none of the members of this class had this subtype). EA = emotional abuse, N = neglect, PA = physical abuse, SA = sexual abuse, SUB1 = 1 subtype of maltreatment, SUB2 = 2 subtypes of maltreatment. Grey shading represents the average in the sample. The non-maltreated class (37.80%) was not represented visually because all item response probabilities are zero for maltreated indicators.

**Figure 2.**
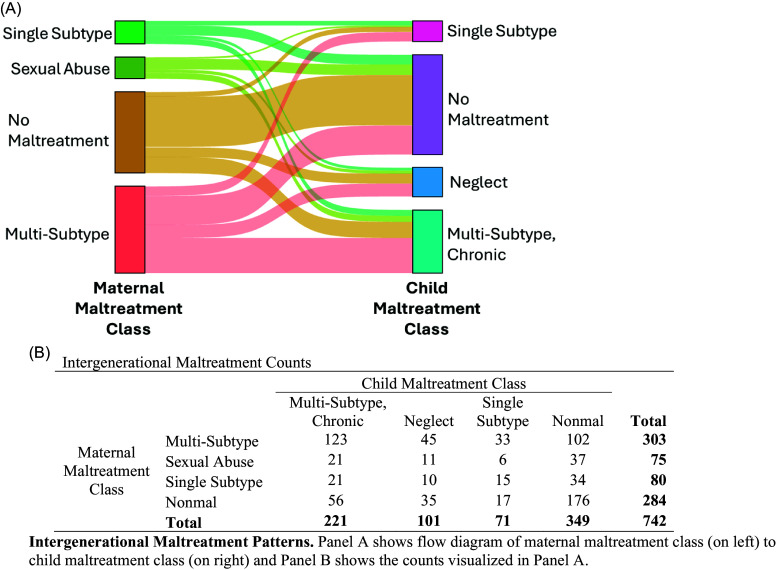
Visual flowchart of intergenerational continuity between four G1 classes and four G2 classes.

**Figure 3.**
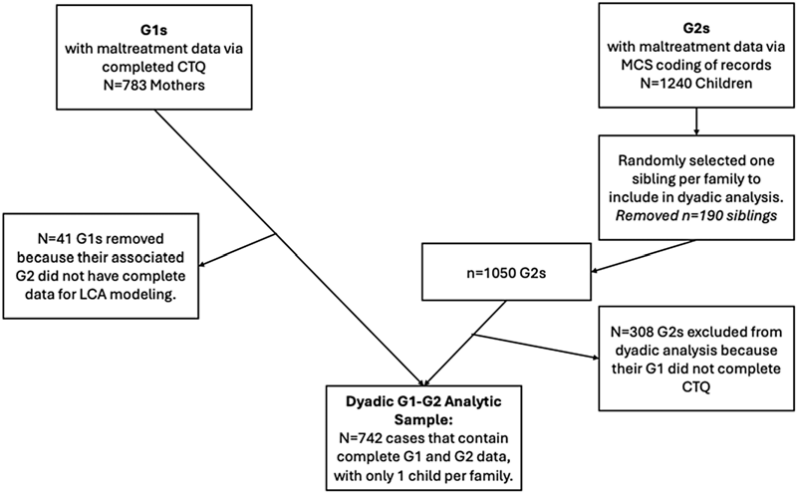
Flow chart of data management and sample size adjustments.

Maltreated children are disproportionately from low-income, single-parent families (USDHHS, 2022). Therefore, the DHS liaison identified demographically comparable families (i.e., families receiving Temporary Assistance for Needy Families) without histories of CPS or preventive services involvement to recruit into the non-maltreated comparison group. As with the maltreated group, the DHS liaison contacted a random sample of eligible non-maltreated participants to discuss study details. If participants expressed interest, then their information was passed to project staff who were provided consent to search family DHS records and further verify the absence of maltreatment for all children in the family. Further, trained research staff conducted the Maternal Child Maltreatment Interview (Cicchetti et al., 2003) with all mothers to confirm the lack of maltreatment. If any conflicting information was provided that suggested the comparison participants may have experienced maltreatment, then they were excluded from the comparison group. Children enrolled in the study participated in a week-long research summer camp and provided assent for research activities. Following their child’s participation in the research summer camp, parents (specifically, biological mothers) completed research interviews in a lab setting that contained a range of interview items intended to assess the mother’s life experiences (e.g., child maltreatment history) and current functioning, as well as their child’s functioning (Figure 1, 2, 3).

To leverage the maximum amount of information available in the data we used all available cases for G1s (*n* = 783) and G2s (*n* = 1240) to derive respective latent classes of G1 and G2 CM exposure. Because G1 visits occurred after G2s attended the research summer camp, some G1s did not complete the visit, which is why the G2 sample is larger than the G1 sample. Following mixture modeling in each respective generation, analysis shifted to tests of intergenerational continuity with G1–G2 dyads, retaining all G1–G2 dyads with complete data in both generations, resulting in a subset of *n* = 742 pairs. See Figure 3 for detailed data management flow chart. G2 participants in this trimmed sample were *M*_age_ = 10.33 (SD = 1.32) and balanced across biological sex (50.1% female). G2 participants were 65.4% Black, 23.4% white, 7.3% bi-racial, 1.1% Asian, 0.5% American Indian/Alaskan Native, and 2.3% “other.” Approximately half of the G2 participants were exposed to maltreatment of any kind (*n* = 393; 53%). This sample trimming process (detailed in Figure 3 flowchart) matches the one used by Negriff (2020) to test intergenerational continuity of latent classes of ACEs in a study of similar design.

### Measures

#### G1 childhood maltreatment exposure

The Childhood Trauma Questionnaire (CTQ; Bernstein et al., 2003) is a widely-used 28-item self-report measure that was completed by mothers to assess history of childhood maltreatment. G1s rated the frequency (1- “never true” to 5- “very often true”) with which they had certain experiences pertaining to childhood maltreatment. Sample items include: “When I was growing up, people in my family hit me so hard it left me with bruises or marks,” “When I was growing up, someone molested me,” and “When I was growing up, people in my family said hurtful or insulting things to me.” The CTQ has evidence of acceptable psychometric properties, including the convergence with childhood trauma interviews (Bernstein et al., 1997). The CTQ scores five subtypes of maltreatment: physical abuse, sexual abuse, emotional abuse, emotional neglect, and physical neglect. Presence or absence of each subtype was determined using established cutoff scores (Bernstein et al., 2003). CTQ subtypes showed good internal consistency in the current sample (*α* range: .76–.97). This self-report instrument was used for the G1 because it was the only source of information about parental exposure to CM.

#### G2 childhood maltreatment exposure

The Maltreatment Classification System (MCS; Barnett et al., 1993) is a comprehensive coding system that can reliably quantify multiple dimensions of CM (e.g., maltreatment subtype, developmental chronicity) from documented records. In this study, it was applied to official CPS records obtained through DHS. MCS reliable coders scored records based on the MCS and determined presence of subtypes for each child in the sample. The MCS identifies four different types of maltreatment (sexual abuse, physical abuse, emotional maltreatment, and neglect). Neglect ratings included lack of supervision, failure to provide, educational neglect, and moral/legal/educational neglect. All four subtypes of maltreatment were included in this study: sexual abuse, physical abuse, emotional maltreatment, and neglect, with “neglect” defined by the presence of any of the four types of neglect listed above. The average intraclass correlations between pairs of coders ranged from .86 – 1.0 for presence of each subtype. Developmental timing of each instance of maltreatment was also scored based on dates of maltreatment experiences. Developmental periods used in this analysis included: infancy (birth – 17 months), toddlerhood (18 months – 2 years), preschool age (3 – 5 years), early school age (6 − 7 years), and later school age (8–12 years). Our specification of developmental periods was empirically- and theoretically-informed by the works of other CM researchers applying a developmental stages framework to the MCS data (e.g., English et al., 2005; Manly et al., 2001; Thornberry et al., 2001), which generally correspond to Eriksonian (1963) psychosocial stages of development.

Maltreatment chronicity was then defined by the number of these developmental periods in which maltreatment was present. There are several meritorious approaches to define chronicity, including counting developmental periods or calendar years in which exposure occurred, as well as determining whether or not there were gaps in the chronicity (i.e., *continuity*; see English et al., 2005 for comparison of approaches). Consistent with other researchers using the MCS, we characterized chronicity based on the number of developmental periods in which CM occurred (i.e., the *developmental definition;* English et al., 2005; Graham et al., 2010; Manly et al., 2001; Thornberry et al., 2001) to adhere to the developmental psychopathology framework that organizes our study. Moreover, there is evidence that this definition of chronicity is a more sensitive predictor of CM sequelae (English et al., 2005; Graham et al., 2010).

### Data analytic plan

An LCA for G1 was conducted with Mplus8 Version 6 (Muthén & Muthén, 2023) using estimation of robust standard errors to account for non-normality of data and full information maximum likelihood to estimate the small amount (*n* = 4) of missing data on indicators. Models were fit from 1 to *k* class solutions until fit statistics did not improve. Comparison of multiple fit indices were used to select the best-fitting class solution. Lower values on Akaike Information Criterion (AIC; Akaike, 1987), Bayesian Information Criterion (BIC; Schwarz, 1978), and adjusted Bayesian Information Criterion (aBIC; Sclove, 1987) indicate a relatively better fitting class solution. However, these comparative fit indices may point to the selection of different models; best practice is to use fit indices in conjunction with other indicators of a stable and replicable class solution (Collins & Lanza, 2009). Higher entropy values indicate greater separation, or distinction, between classes within a solution. A significant Lo-Mendell-Rubin (aLRT) Adjusted Likelihood ratio test and bootstrapped Likelihood ratio test (BLRT) indicates that an *n* class solution is a significantly better fit than the *n-1* model (Collins & Lanza, 2009; Lo et al., 2001). Consistent with recommendations by Collins & Lanza (2009), selection of a best-fitting model depended not only on individual fit indices, but also interpretability of the classes of a solution and empirical identification, or the ability of a given solution to converge on one set of best-fitting parameter estimates. Interpretability of the fit-indicated class solution was based on prevalence of class membership probabilities (percent of the sample that is estimated to belong in a single class) as well as item response probabilities on individual indicators.

#### G1 latent class indicators

For *G1s*, a total of five dichotomous and trichotomous variables describing CM subtype and multiplicity were created from the CTQ. Four variables capturing presence of emotional abuse, physical abuse, sexual abuse, and neglect were used as dichotomous indicators of the latent class solutions. Emotional and physical neglect were collapsed into a single neglect category to reflect the scoring of the MCS. A trichotomous indicator for number of subtypes was also created and included (0 = no maltreatment, 1 = one subtype, 2 = more than 1 subtype) to capture multiple subtype occurrence that is not captured by presence/absence variables for each subtype.

#### G2 latent class analysis

In a series of two papers (Russotti et al., 2025; Warmingham et al., 2019), we identified patterns of maltreatment subtype and chronicity using a 2-step exploratory/confirmatory latent class analysis approach. In these models, indicators included presence/absence of each subtype, multi-type exposure (0, 1, or 2+ subtypes), and chronicity (0, 1, 2+ developmental periods). The multiple subtype indicator was deployed because it can be more discerning in separating patterns of exposure than subtypes alone, improving clarity and interpretability of classes.

We will use the resulting 4-class solution to characterize maltreatment in the G2 sample. This same 4-class characterization of maltreatment exposure has been used in subsequent papers to predict both child and emerging adulthood adaptation and psychopathology, further validating the latent classes as a way to represent meaningful variation in maltreatment exposure (Handley et al., 2021, Warmingham et al., 2023).

#### Intergenerational continuity of CM classes

After the optimal LCA class solution was identified using comparative fit indices in G1s and G2s, respectively, individuals were assigned to their most probable class membership as determined by multiply imputed posterior probabilities for individuals. To examine continuity in intergenerational CM exposure, we first used crosstabulations and chi-square tests to determine if G1’s class membership was associated with their G2’s class membership (analyses conducted in SPSS v29 and Rv4.3.1; data visualization conducted in Rv4.3.1 using fms package (Figure 1) and ggsankey package (Figure 2). We also conducted multinomial logistic regression in Mplus (v8.11) to obtain estimates for how the odds of G2’s CM class change depending on G1’s CM class. In this approach, family-level dependency structure is represented in the model itself (as a path). Parameter estimates, standard errors, odds rations and 95% Confidence Intervals were obtained using robust estimators appropriate for binary observed variables (WLSMV estimator).

## Results

### Descriptives

#### G1 maltreatment exposure

Of the *n* = 783 G1s who completed the CTQ, 62.2% reported maltreatment exposure on the CTQ, with 21.1% exposed to one subtype and 41.1% exposed to two or more subtypes. Prevalence of individual subtype exposure was 33.3% (*n* = 261) for emotional abuse, 33.2% (*n* = 261) for physical abuse, 42.3% (*n* = 331) for sexual abuse, and 35.1% (*n* = 275) for either emotional or physical neglect.

#### G2 maltreatment exposure

Of the *n* = 1240 children who attended the summer research camp, *n* = 593 (47.8%) had families with no involvement with CPS and thus did not have documented maltreatment exposure, and the other *n* = 647 (52.2%) experienced exposure to maltreatment. Of all children included, 30.1% experienced emotional maltreatment, 15.3% experience physical abuse, 4.8% experienced sexual abuse, and 41.9% experienced neglect. Most children with maltreatment exposure (58.1%) were exposed to more than one subtype of maltreatment; 57% of children were exposed to maltreatment in a single developmental period and 42.8% experienced maltreatment in two or more developmental periods.

### Latent class analyses

#### G1 maltreatment

One to seven classes were fit for maternal CM exposure. After review of comparative fit (see Table 1), class sizes, and interpretability of each class solution, we decided the 4-class was the best fitting solution based on a conservative model selection approach. Although the 5-class solution was reasonable, the smallest class did not meet guidelines for minimal class size (i.e., 5% or 50 cases; Collins & Lanza, 2009; Lanza & Rhoades, 2013; Nylund et al., 2007; Nylund-Gibson & Choi, 2018; Muthén & Muthén, 2023; Weller et al., 2020). Given this “edge” decision, we also present the full study results for the 5-class solution in the supplemental materials, including a description of the five classes of G1 maternal CM exposure, as well as the crosstabulations testing the intergenerational associations between the five G1 classes and the four G2 classes.

**Table 1.** Fit information for G1 maternal history of maltreatment latent class analysis

# classes	*n*	# free parameters	LL	AIC	BIC	aBIC	Entropy	aLRT (*p*-value)	BLRT (*p*-value)	Smallest class (%)
1	783	6	−2865.76	5743.52	5771.51	5752.45	–	–	–	–
2	783	13	−2063.68	4153.37	4213.99	4172.71	1.0	<0.001	<0.001	41.12%
3	783	20	−1869.22	3778.44	3871.70	3808.19	1.0	<0.001	<0.001	21.07%
**4**	**783**	**27**	**−1798.88**	**3651.76**	**3777.66**	**3691.92**	**1.0**	**<0.001**	**<0.001**	**10.47%**
5	783	34	−1760.90	3589.81	3748.35	3640.39	1.0	<0.001	<0.001	3.96%
6	783	41	−1740.74	3563.48	3754.67	3624.48	1.0	<0.001	<0.001	1.41%
7	783	48	−1722.79	3541.57	3765.40	3612.98	0.99	0.001	<0.001	3.32%

The 4-class solution was characterized as follows (See Figure 1):

1. Class 1, the *Single Subtype Class* (*n* = 83; 10.60%) was characterized by moderate rates of emotional abuse (13.3%), physical abuse (24.1%), or neglect (62.7%), but not sexual abuse (0%). All members of this class experienced only one subtype of maltreatment.
2. Class 2, the *Sexual Abuse Class* (*n* = 82; 10.47%): All of the members of this class experienced only sexual abuse without exposure to any other subtype.
3. Class 3, the *Multi-Subtype Class* (*n* = 322; 41.12%) was characterized by moderate to high rates of emotional abuse (77.6%), physical abuse (74.5%), sexual abuse (78.1%), and/or neglect (69.3%). All members of this class experienced more than one subtype. This was the most prevalent pattern of maltreatment among mothers.
4. Class 4, the *No Maltreatment Class* (*n* = 296; 37.80%): This class included mothers who did not have any exposure to maltreatment based on CTQ responses (i.e., item response probabilities were 0 for all subtypes).

Described extensively in the supplemental materials, the 5-class solution effectively retains three classes from the 4-class solution (*nonmaltreated, Single Type-Sexual Abuse, and Multi-Type CM*). Distinctly, it appears to further tease apart the *mixed Single Subtype* class from the 4-class solution, which was characterized by moderate rates of emotional abuse, physical abuse, or neglect, but not sexual abuse. The 5-class solution breaks this class down further into two distinct classes: *Single Subtype-Emotional/Physical Abuse Class* and *Single Subtype-Neglect Class*.

#### G2 maltreatment

Maltreatment class characterizations for the G2 sample, as derived in previously published analysis (Russotti et al., 2025 and Warmingham et al., 2019), resulted in 4-class solution, characterized as follows:

1. **Non-maltreatment class (*n* = 593, 47.8%).** Consistent with study design, we identified a group of children with no exposure to maltreatment subtypes.
2. **Single subtype class (*n* = 111, 9.0%).** This pattern of exposure was the least common among children exposed to maltreatment and was characterized by exposure to a single subtype of maltreatment (∼90% ) - either physical abuse (∼40%), emotional maltreatment (∼50%), or sexual abuse (∼12%) that occurred in a single developmental period (∼90% non-chronic maltreatment).
3. **Neglect only class (*n* = 172, 13.9%).** This is the second-largest pattern of maltreatment exposure, and it is characterized by exposure to neglect (100%) that occurred largely in a single developmental period (∼80% single developmental period; ∼20% chronic exposure).
4. **Multi-subtype, chronic maltreatment Class (*n* = 364, 29.4%).** Representing the most common pattern of maltreatment in the G2 sample, this class was typified by multi-subtype exposure (100%). Rates of individual subtypes were ∼ 40% for physical abuse, ∼85% for emotional maltreatment, ∼12% for sexual abuse, and a notable ∼ 95% of children in this class experienced neglect. Exposure tended to be chronic in nature (∼65%).

### Crosstabulations of intergenerational continuity

Results of the 4 (G1 CM classes) X 2 (presence/absence of G2 CM) crosstabulations also revealed that G1 classes of CM exposure were significantly associated with any CM exposure in G2 offspring (*X*^2^ (3) = 46.9, *p* < .001). Notably, 66.3% of G1s in the “*Multi-Type”* exposure class had G2s exposed to CM (adjusted residual = 6.0; OR(vs nonmal) = 3.21, 95%CI: 2.30 – 4.51). G1s in the “*Single-Subtype (No Sexual Abuse)”* had 57.5% of G2s experience maltreatment (adjusted residuals <0.9; OR(vs nonmal) = 2.20, 95%CI = 1.34–3.67). G1s in the “*Single-Subtype (Sexual Abuse)”* class had 50.7% of G2s experience maltreatment (adjusted residuals <0.4; OR(vs nonmal) = 1.67, 95%CI: 1.00–2.80). Similarly, G1s with “*Multi-Type*” CM exposure had significantly fewer G2 children who avoided any CM than would be expected (adjusted residual = −6.0), whereas the other two types of G1 CM exposure were not significantly associated with having G2s avoid CM. Results of 4 X 4 crosstabulation testing the association between the G1 classes of CM exposure and G2 classes of exposure indicate a significant overall association between generations *X*^2^ (9) = 58.42, *p* < .001. *See Figure 2 for visual flowchart of continuity*.

### Multinomial logistic regression

Results of the multilevel multinomial logistic regression demonstrate the relative odds of G2 CM exposure pattern given G1 CM patterning. G1 and G2 reference groups were continuously rotated to obtain all contrasts. See Table 2 for reports of all significant effects. Compared to G1s without CM, G1s with “*Multi-Type*” CM exposure were more likely to have G2s exposed to all forms of CM (v. no exposure). G1s with “*Multi-Type*” CM exposure were most likely (3.79x) to have G2s exposed to “*Chronic, Multi-Type.*” G1s with “*Multi-Type*” CM exposure were also more likely to have G2s exposed to “*Chronic, Multi-Type*” CM exposure than G1s with “*Single-Type (No Sexual Abuse),” or “Single-Type (Sexual Abuse)”* exposure.

**Table 2.** Significant odds ratios

	**G2 CM Class**		
	**Multi, Chronic** (v. No CM)	**Neglect** (v. No CM)	**Single Subtype** (v. No CM)
**G1 CM CLASS**			
**Multi-Subtype** (v. No CM)	3.79 [2.54 – 5.65]	2.22 [1.34 – 3.67]	3.35 [1.78 – 6.32]
**Multi-Subtype** (v. Single Subtype)	1.95 [1.07, 3.57]		
**Multi-Subtype** (v. Sex Abuse)	2.12 [1.17, 3.86]		
**Single Subtype** (v. No CM)	1.94 [1.04 – 3.62]		4.57 [2.08 – 10.02]

	**Multi, Chronic** (v. Single Subtype)	**Single Subtype** (v. Neglect)	**Single Subtype** (v. Multi, Chronic)
**Multi-Subtype** (v. Single Subtype)	2.64 [1.24, 5.73]		
**Single Subtype** (v. No CM)		3.09 [1.15, 8.30]	
**Single Subtype** (v. No CM)			2.35 [1.00, 5.54]

Compared to G1s without CM, G1s with ***“Single-Type (No Sexual Abuse)”*** CM exposure were more likely to have G2s exposed to “*Episodic, Single-Type (Abuse)”* compared to any of the other three G2 CM exposure patterns. G1s with *“Single-Type (No Sexual Abuse)”* CM exposure (v. G1s without CM) were more likely to have G2s exposed to “*Chronic, Multi-Type*” exposure (v. no CM exposure). G1*“****Single-Type (Sexual Abuse)”*** exposure was not significantly associated with any distinct form of G2 CM exposure.

## Discussion

To enhance our understanding of the intergenerational continuity of CM exposure, the current study applied a multivariate, multidimensional approach (Berzenski et al., 2022). First, we used latent class analyses to identify unique, multidimensional patterns of CM exposure in G1s and G2s. We derived four classes of exposure in both generations that were consistent with the extant literature. Second, we determined if certain patterns of G1 CM exposure were more or less likely to result in G2 CM exposure. Thirdly, we examined whether certain multigenerational patterns of CM exposure resulted in homotypic continuity (G1 and G2 share same exposure pattern) or heterotypic continuity (G1 and G2 experience distinct forms of CM). These results are discussed in detail below.

### Latent classes of CM exposure in G1s and G2s

#### G1 classes

We identified four classes of CM exposure in G1s which are consistent with existing literature: a) *Nonmaltreated,* b) *Single-Type (Sexual Abuse-Only),* c) *Single-Type (Non-Sexual Abuse),* d) *Multi-Type Exposure*. Multiple review papers have summarized the results of studies applying LCA methods to retrospective, adult self-reports of CM histories (Debowska et al., 2017; Rivera et al., 2018). Studies have commonly identified 3–4 distinct classes representing subgroups characterized by 1) absence of CM, presence of multi-type CM, presence of sexual abuse only, and a class characterized by the presence of a single, non-sexual abuse exposure (e.g., physical abuse or neglect). Further, these common patterns have been identified in studies specifically examining *maternal* CM (Armour et al., 2014, Guyon-Harris et al., 2021; Hazen et al., 2009; Khoury et al., 2021). Thus, the four classes of CM patterns characterizing the G1s in our sample are consistent with the extant literature.

### G2 classes

Four classes of CM exposure were derived for G2s based on previously published analysis (Russotti et al., 2025; Warmingham et al., 2019*)* and are briefly discussed here. First, a *Nonmaltreated* class characterized by the absence of any CM (*n* = 349, 47%). Second, the most common maltreatment class (>50% of maltreated children)—*Chronic, Multi-Type Exposure—*was characterized by high exposure to neglect in addition to at least one or more other forms of abuse (physical, emotional, or sexual), which occurred chronically in two or more developmental periods (*n* = 221, 29.8%). Third, the *Episodic, Single-Type (Neglect-Only* class included children exposed to neglect, typically in a single developmental period, and represented the second largest class of CM exposure (*n* = 101, 13.6%). Finally, the smallest pattern—*Episodic, Single-Type (Abuse*)—was characterized by a single subtype that was not neglect (i.e., emotional, sexual, or physical abuse) that typically occurred in a single developmental period (*n* = 71, 9.6%).

Compared to G1 classes, there are fewer available studies with which to contextualize our latent classes of G2 CM exposure, given our uniquely rigorous use of MCS-coded, CPS records and inclusion of multidimensional indicators (i.e., all four subtypes, and indicators of polyvictimization and chronicity). Like our findings, Villodas et al (2021), relied on record-based ascertainment of CM exposure and similarly identified four consistent groups: a) nonmaltreated, b) neglect-only, c) abuse-only, and d) multi-type CM. And similar to Ziobrowski et al (2020), we identified a multi-type exposure pattern that occurred across multiple developmental periods.

The combination of multi-type and chronic CM exposure in G2s is notable, and the etiology is likely multifaceted. One explanation is that the severity and complexity of experiencing multiple types of CM can lead to cumulative risk and compounding vulnerabilities that escalate into enduring, chronic CM (Cicchetti & Toth, 2005; Dodge et al., 1990; Herrekohl & Herrenkohl, 2007). Further, in the presence of multi-type exposure, child welfare and preventive systems may fail to intervene across all forms of CM occurring, allowing CM to persist in some form or another. Likewise, CM exposure can progressively isolate survivors, preventing access to the resources and individuals that could protect against continued exposure (Noll, 2021). Finally, chronic CM tends to be an indicator of larger dysfunction within the family system (e.g., substance use, psychopathology, violence, instability), increasing the likelihood a child is exposed to multiple forms of CM, and ultimately creating environmental conditions where CM becomes routine and entrenched (Belsky, 1993; Cicchetti & Rizley, 1981; Widom, 1999).

### Comparing G1 and G2 classes

We identified consistent patterns across generations, such that G1s and G2s both had a no exposure class, a multi-type exposure class, and two distinct single-type exposure classes. However, due to our study design (i.e., different measurement of CM and slightly different LCA indicators between G1s and G2s), G1 and G2 classes were not mirror images. For example, we were unable to include an indicator of chronicity for G1s because the CTQ does not assess the developmental timing of exposure. For G2s, we know patterns of multi-type exposure were also typically chronic in nature, whereas we cannot be sure the same was true of G1s with multi-type exposure.

Additionally, although we identified two respective “single-type exposure” classes for both G1s and G2s, there were nuanced differences across generations. In G1s, we extracted a “sexual abuse-only” class and another single-type class that was characterized by the presence of neglect (most prominently) or emotional or physical abuse, but *not* sexual abuse. This is contrasted with G2s, where we identified two classes more clearly differentiated by abuse-exposure (emotional, physical, or sexual abuse hanging together) vs neglect-exposure.

One explanation for the differences is that the G1s were all female participants, whereas G2s were a split of male and female participants, which may have increased the likelihood we would identify a “sexual abuse-only” class in G1s. Although previous research suggests that common latent patterns of CM exposure tend to be invariant across biological sex (Rivera et al., 2018), some studies have demonstrated that females are more likely to be classified into sexual-abuse only classes (Armour et al., 2014). Indeed, the presence of a “sexual abuse-only” class is consistently identified in the extant literature on latent classes of *maternal* CM exposure (Armour et al., 2014, Guyon-Harris et al., 2021; Hazen et al., 2009; Khoury et al., 2021).

Another explanation for class differences is the varying developmental range assessed for CM exposure. G1s were asked retrospectively for any exposures occurring from ages 0–18, whereas G2 children were assessed for exposure between ages 0–12. Sexual abuse is more prevalent in later childhood/adolescence (Trickett et al., 2011), which may explain why G1s contained a “sexual abuse-only” class. Conversely, neglect that does not co-occur with abuse is more typical in earlier developmental periods, which may explain the “neglect-only” class identified among G2s. Lastly, while it is common for intergenerational studies to use self-reports for G1 exposure and records for G2 exposure (e.g., St-Laurent et al., 2019; Widom et al., 2015; Yang et al., 2018), certain forms of CM may be more prevalent when exposure is ascertained via retrospective self-report vs official records, possibly contributing to the slight variation in single-type exposure classes in G1 and G2s, respectively.

### (Dis)Continuity in patterns of CM exposure

Adopting a multivariate (i.e., latent class), multidimensional view of intergenerational continuity allows for several promising questions to be answered (Berzenski et al., 2022). First, we can examine whether certain forms/patterns of maternal CM may be more vulnerable to intergenerational continuity in general (i.e., *undifferentiated* approach; Berzenski et al., 2022). For instance, consistent with previous findings (see Madigan et al., 2019 for review), we demonstrate intergenerational continuity of CM exposure. However, our multivariate, multidimensional approach reveals more nuanced detail. Our results suggest that continuity is largely driven by a subgroup of mothers exposed to a pattern of CM marked by multi-type exposure. G1s in the “multi-type exposure” class had significantly more G2s who were exposed to any CM than G1s with other forms of CM exposure. This finding is consistent with other studies demonstrating that certain characteristics of G1’s CM exposure influence intergenerational continuity (Ben-David et al., 2015; Pears & Capaldi, 2001; Thornberry & Henry, 2013), with multi-type exposure acting as a particularly strong predictor of continuity (Bartlett & Easterbrooks, 2015; Jaffee et al., 2013; McKenzie et al., 2024; St-Laurent et al., 2019). Notably, previous studies in this line of inquiry have applied a univariate approach to modeling dimensions of G1 exposure, typically examining how a single dimension (e.g., severity, type, or multiplicity) affects undifferentiated continuity. Our findings advance this line of research by simultaneously considering multiple dimensions of G1 CM exposure (i.e., indicators of all four subtypes, as well as multi-type exposure).

### Differentiated approach to continuity

We also apply a *differentiated* approach to intergenerational continuity by examining how certain multidimensional patterns of CM exposure in G1s are more likely to lead to unique multidimensional patterns of CM exposure in G2s. Thus, we can determine if there is homotypic or heterotypic continuity in multidimensional patterning of CM exposure. As no previous study has investigated concordance between mother and offspring CM patterning, the present study adds to our knowledge of the prevalence and type of intergenerational continuity. Perhaps most importantly, results reveal the presence of homotypic continuity of multi-type CM exposure. G1s in the *multi-type* exposure class were likely to have G2s in the *chronic, multi-type* exposure class (approximately 40% of mothers with multi-type exposure had children with *chronic, multi-type* exposure). There were two important findings for G1s with multi-type exposure: 1) the most likely outcome for their G2 was exposure to chronic, multi-type, and 2) these mothers were more likely than all other G1s to have G2s with chronic, multi-type exposure. This suggests that not only is multi-type exposure more likely to result in undifferentiated continuity of CM, but it is also likely to result in a severe form of exposure (chronic, multi-type) that has been linked to more deleterious outcomes (e.g., Warmingham et al., 2019).

We can briefly speculate on why multi-type CM exposure may continue across generations. The most parsimonious explanation is that multi-type CM exposure represents a broader CM experience, which results in broader consequences for G1s, which beget broader CM risk for G2s. Child maltreatment is multiply determined (Belsky, 1993; Cicchetti & Rizley, 1981) and multi-type exposure results in a more pervasive and detrimental sequelae that permeates and spreads across multiple domains of functioning (Cicchetti & Toth, 2016; Widom, 2022). Therefore, G1s with multi-subtype exposure may carry a more *generalized* risk to perpetrate multiple forms of CM (i.e., intergenerational transmission) or unintentionally recreate environments of multifaceted CM risk for their children—or both—increasing the likelihood G2s would experience multiple forms of CM in a chronic pattern. In essence, additional forms of CM serve as a multiplying force for intergenerational risk. Conversely, exposure to singular, specific forms of CM may yield a more contained, unique pattern of sequelae that confers *specific* CM risk for offspring, resulting in single-type exposure (Font et al., 2020; Noll, 2021).

The G1 “Single-Type (No Sexual Abuse)” exposure class was the only other class exhibiting signs of continuity, though interpreting the form is less straightforward due to the mixed characterization of this group (mothers in this group had single-type exposure to one of emotional abuse, physical abuse, or neglect). G1s with this pattern of CM had more G2s with “Single-Type (Abuse)” exposure. Thus, some dyads in this pairing may have exhibited homotypic continuity (e.g., singular experiences of physical abuse or emotional abuse), whereas others may have had heterotypic continuity (e.g., G1 neglect to G2 physical abuse, G1 emotional abuse to G2 physical abuse).

### Implications

In sum, our findings advance the multidimensional study of intergenerational CM continuity in several ways. First, consistent with other studies, our results support the notion that homotypic continuity of CM is more common than heterotypic continuity (Berzenski et al., 2022). But importantly, we demonstrate that this is true of *multidimensional patterns of CM exposure*, not only of univariate parameters. Additionally, given the equivocal findings in the extant literature on intergenerational CM continuity, we provide greater clarity by highlighting a specific form of exposure (i.e., multi-type exposure) that is linked across generations and may be implicated in intergenerational risk.

Further, despite a strong imperative to provide preventive interventions to families, existing preventive interventions have not been particularly effective in reducing intergenerational continuity of CM (Hart et al., 2024; Viswanathan et al., 2024; Whitcombe-Dobbs & Tarren-Sweeney, 2019; van IJzendoorn et al., 2020). One contributing factor is the reality that parents who experience CM are a heterogenous group with differing needs, and preventive interventions lack tailoring Whitcombe-Dobbs & Tarren-Sweeney, 2019). Indeed, the mechanisms by which G1 CM influences the prevalence and form of CM in G2s may vary across different forms of CM (Berzenski et al., 2022; Langevin et al., 2023; Langevin et al., 2021; McKenzie et al., 2021). Certain mechanisms may be unique to the continuity of certain patterns of CM, while other mechanisms may be universally involved in continuity; and even when universal, some mechanisms may operate differently depending on which forms of CM they are maintaining (see Berzenski et al., 2022). For example, maybe maternal substance use links *multi-type exposure* in G1s to *chronic, multi-type* exposure in G2s, whereas unstable, unhealthy adult relationships maintain *single-type abuse* patterns across generations. Continued efforts to elucidate mechanisms linking multidimensional patterns of CM across generations will elevate our capacity to deliver targeted interventions.

Moreover, the current study established the initial presence of homotypic continuity of *multi-type* exposure. While all CM is harmful, *multi-type* exposure, and especially *chronic, multi-type* exposure, is profoundly detrimental (e.g., Font & Maguire-Jack, 2020; Jonson-Reid et al., 2012; National Research Council, 2014). Indeed, the economic costs of CM are driven upward by this relatively small segment of the overall population that comes in contact with the child welfare system repeatedly (Kim & Drake, 2019). Our findings suggest that one way to prevent chronic, multi-type CM is to better understand and address the CM history *of the parent* as part of secondary and tertiary intervention efforts, with a specific focus on the *patterning of the parent’s CM exposure*. Parents with multi-type CM histories represent a critical target for intervention and should be considered a priority for secondary/tertiary prevention services when child welfare services are limited (as is so often the case). Further, the design of preventive interventions for parents with multi-type CM histories should be broad and multidimensional to best ameliorate the intergenerational consequences. The sequelae of multi-type CM exposure is likely to pervasively balloon across multiple domains of functioning (i.e., psychological, emotional, interpersonal, romantic, socioeconomic, etc.), which in turn may strain the family system, depleting resources and introducing greater level of risk for chronic, multi-type CM exposure in offspring. Thus, secondary/tertiary preventive interventions to support these parents should be equally comprehensive to address the multifaceted sequelae and stabilize families earlier in the cycle.

If our results are conclusive, future studies can advance our understanding of this particular form of intergenerational continuity by investigating mechanisms that determine whether mothers with multi-type CM exposure will *maintain cycles* (have offspring with chronic, multi-type exposure) or *break cycles* (have offspring who evade CM), as well as situations where mothers who did not experience CM go on to have offspring who experience chronic, multi-type exposure (*cycle initiators*). Such studies can improve the precision of our secondary prevention efforts for a particularly high-risk subgroup.

### Strengths and limitations

Current findings should be interpreted within the context of study limitations. A primary limitation is the use of discrepant assessments of CM exposure in G1 and G2 participants. Retrospective self-reports and coded records may reveal different information and may not be directly comparable. That said, the use of discrepant measures is a common trait of almost all intergenerational continuity studies (Bartlett & Easterbrooks, 2015; Islam et al., 2023; Kim, 2009; St-Laurent et al., 2019; Yang et al., 2018; c.f., McKenzie et al., 2021; Widom et al., 2015). Also, by design, half of our G2 participants were recruited for CM histories, which may over-estimate the prevalence of intergenerational risk. This is typically more concerning when studying intergenerational *transmission,* or undifferentiated continuity (Berzenski et al., 2022; Widom, 2015), whereas our study is focused on heterogeneity within maltreated groups. Consistent with most empirical studies of intergenerational continuity (Madigan et al., 2019), our study focused on G1 mothers, specifically, and studies with other caregivers (e.g., fathers) is warranted. Additionally, we elected to operationalize the chronicity and multi-type indicators as categorical variables in order to perform an LCA and match the larger person-centered CM literature (Debowska et al., 2017; Rivera et al., 2018); however, a latent profile analysis, which considers dimensional (vs categorical) measured indicators of maltreatment, would be an alternative approach. Given the well-recognized heterogeneity of neglect (Dubowitz et al., 2022; Ogle et al., 2022), our decision to “lump” emotional and physical neglect into a singular “neglect” indicator may obfuscate important differences in CM patterns that would be informative for understanding (dis)continuity in intergenerational CM exposure. Future studies may consider a more granular approach to intergenerational continuity by “dividing” subcategories of CM subtypes and testing for homotypic/heterotypic continuity. Finally, G1 and G2 maltreatment experiences are not fully independent, as both individuals belong to the same family and share common contextual influences. Families in the dataset are highly homogenous due to the rigorous demographic matching implemented in the research design, however, we could not account for all family-level variance. Future studies may consider applying alternative study designs and use new person-centered methods for modeling family-level processes over time (e.g., RI-LTA; Nylund-Gibson et al., 2023).

The present study has several strengths that offset the limitations. This study includes a rigorous operationalization of CM in G2s through systematically-coded CPS records. Further, our study relied on multivariate, multidimensional indicators to operationalize CM, including markers of four subtypes, polyvictimization, and chronicity. Many other studies on intergenerational continuity assess only certain subtypes of CM, or exclude other characteristics (e.g., chronicity). Another strength of this study is the inclusion of a counterfactual (nonmaltreated) group that was demographically-matched by design. Thus, we were able to obtain CM exposure data for both the maltreated and comparison groups, which can better approximate the prevalence of intergenerational continuity patterns for community families and child welfare involved families. Additionally, our large, unique sample size improves confidence in our findings and their generalizability. Lastly, and importantly, our approach to testing intergenerational continuity is completely novel and innovative, relying on multivariate statistical methods (i.e., latent class analyses) and applying a differentiated, multidimensional view of continuity (Berzenski et al., 2022).

## Conclusion

In this study, we applied multivariate statistics (i.e., latent class analyses) and a multidimensional paradigm (Berzenski et al., 2022) to the study of intergenerational continuity of CM to facilitate greater specificity in our understanding of how certain forms of CM exposure cross generations. The results of the current study point to the presence of intergenerational continuity of CM and clarify that continuity may be largely attributable to a certain form of CM exposure—multi-type exposure. Further, we demonstrate that multi-type exposure in one generation is likely to beget chronic, multi-type exposure in the next generation (homotypic continuity). Thus, the effects of CM reverberate across generations, and our primary goal should be to prevent CM before it occurs through secondary prevention interventions that can interrupt intergenerational cycles. A deeper understanding of the heterogeneity inherent in intergenerational continuity of CM can serve as a critical step toward better informing those interventions with precision.

## Supporting information

10.1017/S0954579425100217.sm001 Russotti et al. supplementary material Russotti et al. supplementary material

## Data Availability

Data is not available due to the inclusion of information obtained from Child Protective Service (CPS) records and data sharing restrictions that were part of the data agreement with governing bodies.

## Supplementary material

The supplementary material for this article can be found at https://doi.org/10.1017/S0954579425100217.
