# Supplementary material for: Intergenerational continuity of multidimensional patterns of child maltreatment exposure: A person-centered approach
Source: Dev Psychopathol. 2025 Jul 3:1–13. Online ahead of print. doi: 10.1017/S0954579425100217 (PMC12233131; doi:10.1017/S0954579425100217)
Supplement: Russotti et al. supplementary material [file S0954579425100217sup001.docx]

Supplemental Materials

**Results based on 5-class solution for G1 CM**

***Latent Class Analyses***

One to seven classes were fit for G1 CM exposure. As noted in the manuscript, both the 4-class and 5-class solutions were reasonable fits for the data based on information criteria indices. However, the 4-class solution was ultimately selected because it provided a more parsimonious and replicable class structure. Although there are no explicit absolute thresholds for minimum class size, the general rule-of-thumb is that class solutions with classes representing less than 5% of the sample (or classes containing fewer than 50 cases) are less stable and reproducible (e.g., Collins & Lanza, 2010; Lanza & Rhoades, 2013; Nylund et al., 2007; Weller et al., 2020). In our study, the 5-class solution yielded three classes at 10% or less, with the smallest class not meeting guidelines for minimum class size (3.96%, n = 31). We elected for the more conservative approach in the manuscript, but given the “edge” decision, we also present the study results for the 5-class solution, including a description of the five classes of G1 maternal CM exposure, as well as the crosstabulations testing the intergenerational associations between the five G1 classes and the four G2 classes.

The 5-class solution for G1 maternal CM exposure was characterized as follows:

1. *No Maltreatment Class* (n = 296; 37.80%): This class included mothers who did not have any exposure to maltreatment based on CTQ responses (i.e., item response probabilities were 0 for all subtypes).
2. *Multi-Subtype Class* (n = 322; 41.12%) was characterized by moderate to high rates of emotional abuse (77.6%), physical abuse (74.5%), sexual abuse (78.1%), and/or neglect (69.3%). All members of this class experienced more than one subtype. This was the most prevalent pattern of maltreatment among mothers.
3. *Sexual Abuse Class* (n = 82; 10.47%): All of the members of this class experienced only sexual abuse without exposure to any other subtype.
4. *Single Subtype-Emotional/Physical Abuse Class* (n = 31; 3.96%): All of the members of this class experienced only a single type of abuse, either emotional (0.36) or physical abuse (0.65).
5. *Single Subtype-Neglect Class* n = 52; 6.64%): All of the members of this class experienced only neglect without exposure to any other type.

Below are the item response probabilities for the 5-class solution:

|  | Class 1 (41.1%) | Class 2 (4.0%) | Class 3 (6.6%) | Class 4 (10.5%) | Class 5 (37.8%) |
| --- | --- | --- | --- | --- | --- |
| Emotional Abuse | 0.78 | 0.36 | 0.0 | 0.0 | 0.0 |
| Physical Abuse | 0.75 | 0.65 | 0.0 | 0.0 | 0.0 |
| Sexual Abuse | 0.78 | 0.0 | 0.0 | 1.0 | 0.0 |
| Neglect | 0.69 | 0.0 | 1.0 | 0.0 | 0.0 |
| # Subtypes |  |  |  |  |  |
| *0* | 0.0 | 0.0 | 0.0 | 0.0 | 1.0 |
| *1* | 0.0 | 1.0 | 1.0 | 1.0 | 0.0 |
| *2+* | 1.0 | 0.0 | 0.0 | 0.0 | 0.0 |

**Comparison to the 4-class results.** The 5-class solution effectively retains three classes from the 4-class solution (*nonmaltreated, Single Type-Sexual Abuse, and Multi-Type CM*). Distinctly, it appears to further tease apart the *mixed Single Subtype* class from the 4-class solution (n=83, 10.6%), which was characterized by moderate rates of emotional abuse (13.3%), physical abuse (24.1%), or neglect (62.7%), but not sexual abuse (0%). The 5-class solution breaks this class down further into two distinct classes: *Single Subtype-Emotional/Physical Abuse Class* and *Single Subtype-Neglect Class*.

***Intergenerational continuity of CM classes***

G1s and G2s were assigned to their most probable class membership as determined by assigning most probable class membership based on highest posterior probabilities for individuals. To examine continuity in intergenerational CM exposure we used crosstabulations and chi-square tests to determine if G1’s class membership was associated with their G2’s class membership (analyses conducted in SPSS v29). When probing sources of significance within the 4x4 crosstabulations, adjusted residual values >2 were considered to be of significance.

**5 x 4 crosstabulation.** Results of 5 X 4 crosstabulation testing the association between the G1 classes of CM exposure and G2 classes of exposure indicate a significant overall association between generations *X*^2^ (12) = 61.6, p < .001. Of G1s with “*Multi-Type*” CM exposure, 40.6% had G2s exposed to “*Chronic, Multi-Type*” CM exposure (adjusted residual = 5.3). G1s with “*Multi-Type*” CM were not significantly more likely to have G2s experience other forms of CM such as “*Episodic, Single-Type (Neglect)”* (adjusted residual = 0.8) or “*Episodic, Single-Type (Abuse)”* (adjusted residual = 1.0).

G1*“Single-Type (Sexual Abuse)”* exposure was not significantly associated with any distinct form of G2 CM exposure: “*Chronic, Multi-Type*” (adjusted residual = -0.4), “*Episodic, Single-Type (Neglect)”* (adjusted residual = 0.3), or “*Episodic, Single-Type (Abuse)”* (adjusted residual= -0.5).

G1s with “*Single-Type (EA/PA)”* exposure had significantly more G2s who were exposed to “*Episodic, Single-Type (Abuse)”* exposure (adjusted residual = 2.0). However, G1 “*Single-Type (EA/PA)”* was not associated with G2 “*Chronic, Multi-Type*” exposure (adjusted residual = 0.4) or “*Episodic, Single-Type (Neglect)”* (adjusted residual = 0.0) exposure.

G1s with “*Single-Type (Neglect)”* exposure had significantly more G2s who were exposed to “*Episodic, Single-Type (Abuse)”* exposure (adjusted residual = 2.1). However, G1 “*Single-Type (Neglect)”* was not associated with G2 “*Chronic, Multi-Type*” exposure (adjusted residual = -1.2) or “*Episodic, Single-Type (Neglect)”* (adjusted residual = -0.3) exposure.

G1s without CM exposure (i.e., *nonmaltreated*) had significantly fewer G2s exposed to “*Chronic, Multi-Type*” CM (adjusted residual = -4.7) and “Episodic, Single-Type (Abuse)” CM (adjusted residual = -2.6). However, a lack of CM exposure in G1s was not significantly associated with G2 exposure to “*Episodic, Single-Type (Neglect)”* CM (adjusted residual = -0.8).

**Comparison to 4-class results.** Given that three of the G1CM classes remained the same between the 4- and 5-class solutions, the only novel results pertain to the two distinct classes that emerge from the 5-class solution: *Single Subtype-Emotional/Physical Abuse Class* and *Single Subtype-Neglect Class*. Both classes resulted in the same intergenerational associations as the larger, 4-class solution class from which they were derived (*Single Type-No Sexual Abuse)*. Specifically, G1 *Single Subtype (Emotional/Physical Abuse)* exposure and *Single Subtype (Neglect)* exposure was significantly associated with G2 *Episodic, Single-Type (Abuse)* exposure, but were not significantly associated with any other pattern of G2 exposure.
